# Durable Response After Combination Therapy With Enfortumab Vedotin and Radiotherapy in Metastatic Urothelial Carcinoma: A Report of Two Cases

**DOI:** 10.7759/cureus.49936

**Published:** 2023-12-04

**Authors:** Daiki Ikarashi, Koyo Kikuchi, Kenta Takahashi, Hisanori Ariga, Wataru Obara

**Affiliations:** 1 Urology, Iwate Medical University Hospital, Iwate, JPN; 2 Radiation Oncology, Iwate Medical University Hospital, Iwate, JPN

**Keywords:** tumor shrinkage, durable response, urothelial carcinoma, radiation, enfortumab vedotin

## Abstract

Enfortumab vedotin for urothelial carcinoma is a potentially effective anti-tumor drug that can be used in 3rd-line therapy or later, even in relatively advanced stages of the disease. Here, we present two cases of treatment using enfortumab vedotin with subsequent radiotherapy for primary lesions, and long-term disease control was achieved. The first case involved a 78-year-old man previously treated with pembrolizumab following gemcitabine plus carboplatin for lower ureteral carcinoma with multiple lung and lymph node metastases. Six months after the initiation of enfortumab vedotin, the primary tumor and metastases notably shrank. However, the primary tumor regrew, and radiotherapy was initiated along with enfortumab vedotin. The second case involved a 60-year-old man who was initially treated with avelumab following gemcitabine plus cisplatin for bladder cancer with multiple lymph node metastases. After two months of enfortumab vedotin, the primary and metastatic lesions shrunk. However, the primary tumor regrew, and radiotherapy was initiated. In both cases, the primary tumor and metastases recorded long-term shrinkage. The combination of radiotherapy and enfortumab vedotin may be an effective treatment option.

## Introduction

Enfortumab vedotin (EV) is a new antibody-drug conjugate that combines a human anti-nectin-4 antibody with a highly potent microtubule disruptor, monomethyl auristatin E (MMAE) [1]. EV therapy for locally advanced or metastatic urothelial carcinoma (UC) in the EV-301 trial showed objective and complete response rates of 40.6% and 4.9%, respectively [2]. The trial included patients who experienced disease progression during or after treatment with immune checkpoint inhibitors following prior treatment with platinum-based chemotherapy [2]. Although the response rate of EV is high from the third-line treatment or later [3], the progression-free and overall survivals are limited to 5.5 months and 12.8 months, respectively [2], indicating the absence of a long-term response. Hence, new treatment strategies aimed at long-term response need to be explored. Indeed, the exploration of combination therapy with EV and other antitumor agents, such as immune checkpoint inhibitors, has progressed recently [4]. However, the effectiveness of EV plus radiotherapy remains unclear. Here, we demonstrated a successful, durable response to radiotherapy with EV for metastatic UC.

## Case presentation

Case 1

**Figure 1:**
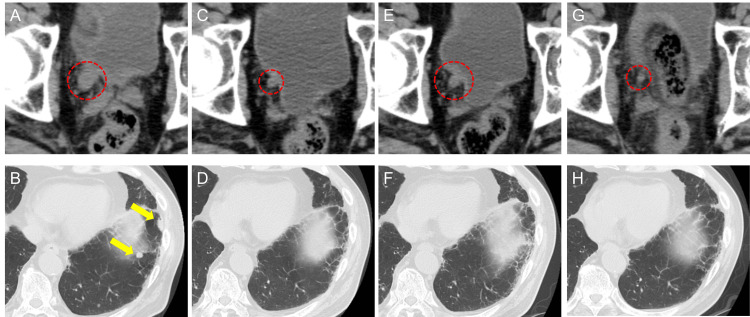
Durable response to enfortumab vedotin and additional radiation therapy in Case 1 Computed tomography (CT) showing shrinkage and increase of the primary right ureteral carcinoma (red circle) and lung metastasis (yellow arrow) in case 1. (A, B) Pretreatment; (C, D) after six cycles; and (E, F) after nine cycles of enfortumab vedotin (EV) therapy. (G, H) After additional radiation with EV therapy, maintenance of tumor shrinkage for the primary ureteral carcinoma and lung metastasis was noted.

A 78-year-old man with a history of three transurethral resections of bladder tumor and intravesical Bacillus Calmette-Guerin for non-muscle-invasive bladder cancer presented to our hospital with macrohematuria. He had a history of diabetes, chronic renal failure, and allergy to contrast media. CT showed right lower ureteral carcinoma with hydronephrosis. A ureteroscopic biopsy led to a pathological diagnosis of high-grade (G2), locally advanced ureteral carcinoma with a clinical diagnosis of cT3N0M0. Initially, gemcitabine plus carboplatin was initiated as neoadjuvant chemotherapy. After three cycles of chemotherapy, the primary tumor became enlarged, and a right obturator lymph node appeared. As such, pembrolizumab was administered as second-line therapy. However, after eight cycles of pembrolizumab, the primary tumor became enlarged, and new lung metastases appeared; the patient was administered EV as third-line therapy (Figures 1a, 1b). EV was started at 1.25 mg/kg. After six cycles of EV, the primary ureteral carcinoma shrank from 13 to 6 mm, and the lung metastases almost disappeared (Figures 1c, 1d). However, after nine cycles of EV, although the metastatic lesions remained shrunken, the primary ureteral carcinoma slightly increased from 6 to 8 mm (Figures 1e, 1f). Since the primary ureteral carcinoma was expected to grow, we recommended radiotherapy for the primary lesion based on a joint urology and radiology conference. He started radiation (55 Gy/20 Fr) for the primary ureteral carcinoma while continuing EV therapy. There were no radiation-related adverse events, and tumor shrinkage was maintained in both primary and metastatic lesions for 16 months from the initiation of EV therapy (Figures 1g, 1h).

Case 2

**Figure 2:**
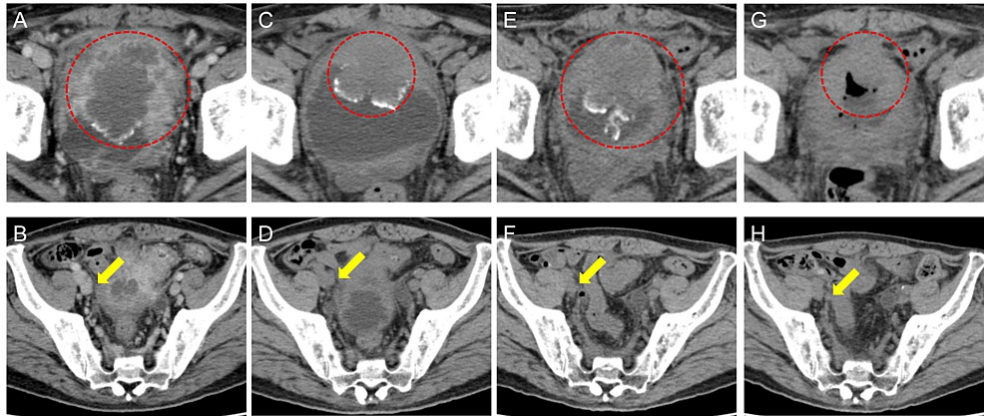
Durable response to enfortumab vedotin and additional radiation therapy in Case 2 Computed tomography (CT) showing shrinkage and increase of the primary bladder tumor (red circle) and lymph node metastasis (yellow arrow) in case 2. (A, B) Pretreatment; (C, D) after two cycles; and (E, F) after three cycles of enfortumab vedotin (EV) therapy. (G, H) After additional radiation with EV therapy, indicating maintenance of tumor shrinkage for the primary ureteral carcinoma and lymph node metastasis was noted.

A 60-year-old man presented with macrohematuria. He had no medical history. Cystoscopy showed a wide-base nonpapillary tumor at the bladder apex. The pathological diagnosis was high-grade (G2) UC. MRI and CT showed invasive bladder cancer and multiple pelvic lymph node metastases (cT3N2M0). The patient started gemcitabine plus cisplatin therapy as first-line treatment. After four cycles of chemotherapy, there was no change in tumor size, as the patient had stable disease. Therefore, he was introduced to avelumab as maintenance therapy. However, after eight cycles of avelumab, tumor growth was observed in both the primary and metastatic sites, and the patient was administered EV as third-line therapy (Figures 2a, 2b). EV was started at 1.0 mg/kg because of a skin rash in the previous treatment. After two EV cycles, the primary bladder tumor shrank from 72 to 46 mm, and lymph node metastases almost disappeared (Figures 2c, 2d). However, after three EV cycles, although the metastatic lesions remained shrunken, the primary bladder tumor slightly increased in size from 46 to 55 mm (Figures 2e, 2f). We then started radiation (36 Gy/6 Fr) to the primary bladder tumor. The patient continued EV therapy without radiation-related adverse events, and tumor shrinkage was maintained in both primary and metastatic lesions for 12 months from the initiation of EV therapy (Figures 2g, 2h).

## Discussion

We successfully demonstrated two cases treated with EV and radiotherapy, which provided durable control of primary and metastatic lesions. These cases highlight important clinical points, including the effects of irradiation on sites that have acquired resistance to EV.

The mechanism of resistance to EV therapy is due to several mechanisms, such as antigen-related resistance, failure of internalization, impaired lysosomal function, and others [5]. Among these, nectin-4 expression in target lesions associated with antigen-related resistance was one of the most important factors in the effectiveness of EV [6]. Furthermore, previous studies reported that nectin-4 expression changed with disease progression to metastatic disease [6,7]. Although a 59.1% decrease in nectin-4 expression was observed in the metastatic biopsy, it showed a 19.1% increase relative to the nectin-4 expression in the primary lesion, indicating the difference in EV efficacy between primary and metastatic lesions [7]. Namiki et al. reported that the tumor shrinkage effect differed by metastatic sites treated with EV therapy [8]. They also suggested that treatment strategies to overcome resistance to EV are required.

The anti-tubulin agent MMAE, as the key drug of EV, has potent radiosensitization. MMAE radiosensitization showed both schedule and dose dependency, directly correlating with the accumulation of cells in the G2/M checkpoint [9]. Chemotherapy drugs such as paclitaxel, an anti-tubulin agent, induce a strong arrest of cells in the G2/M phase of the cell cycle as radiosensitizers and have been used along with radiotherapy in real-world clinical practice [10]. The patients in this case were treated with daily irradiation of 55 Gy/20 Fr and weekly irradiation of 36 Gy/6 Fr without adverse events. The efficacy and safety of radiotherapy for bladder cancer according to each treatment schedule have been established in randomized controlled trials [11-13]. These results suggested that combining radiotherapy of the primary lesion with EV therapy might be an effective treatment option for UC with metastases under disease control.

A limitation of this case series was that conclusions could not be drawn about the efficacy and safety of combined radiotherapy and EV therapy. Further clinical research with larger sample sizes and more comprehensive methodologies would be needed to validate these findings. In addition, we did not perform biopsies prior to EV administration. Therefore, we were unable to confirm nectin-4 expression in the primary and metastatic lesions. However, we believe that the clinical information presented in this case series can help clarify the potential therapeutic spectrum of combined radiotherapy and EV therapy for metastatic UC.

## Conclusions

We showed two cases of durable response to radiotherapy and EV therapy for metastatic UC. Although the durability of tumor shrinkage in EV therapy is limited due to resistance mechanisms, long-term disease control may be possible with combined local irradiation. We suggest that adding radiotherapy to EV therapy might be an effective treatment option for patients with UC who have metastases with controlled disease.
